# Developmental dynamics of homoarginine, ADMA and SDMA plasma levels from birth to adolescence

**DOI:** 10.1007/s00726-023-03318-w

**Published:** 2023-08-30

**Authors:** Florence Baach, Boglarka Meyer, Jun Oh, Susanne Lezius, Rainer Böger, Edzard Schwedhelm, Chi-un Choe, Axel Neu

**Affiliations:** 1 Department of Paediatrics, University Medical Center Hamburg-Eppendorf, Martinistraße 52, 20246 Hamburg, Germany; 2 Department of Medical Biometry and Epidemiology, University Medical Center Hamburg-Eppendorf, Hamburg, Germany; 3 Institute of Clinical Pharmacology and Toxicology, University Medical Center Hamburg-Eppendorf, Hamburg, Germany; 4 German Centre for Cardiovascular Research (DZHK E.V.), Partner Site Hamburg/Kiel/Lübeck, Hamburg, Germany; 5 Department of Neurology, University Medical Center Hamburg-Eppendorf, Hamburg, Germany; 6 Present Address: Department of Neurology, Klinikum Itzehoe, Robert-Koch-Strasse 2, 25524 Itzehoe, Germany; 7 Present Address: VAMED Klinik Geesthacht, Johannes-Ritter-Strasse 100, 21502 Geesthacht, Germany

**Keywords:** Paediatric, L-arginine, Guanidino compound

## Abstract

Guanidino compounds such as dimethylarginines (SDMA, ADMA) and L-homoarginine ((L-)hArg) can interfere with bioavailability and function of the main NO-donor L-arginine (L-Arg). High ADMA and SDMA but low L-hArg concentrations have been associated with cardio- and cerebrovascular events and mortality in adults. The role of guanidino compounds in paediatric patients remains less clear. We, therefore, compared guanidino compound levels in plasma samples of 57 individuals with chronic kidney disease (CKD) and 141 individuals without CKD from the age of 0 to 17 years, including patients with different comorbidities by correlation and regression analyses. We found highest hArg, SDMA and ADMA concentrations in neonates (Kruskal–Wallis, *p* < 0.001 for all). From the age of 1 year on, hArg levels increased, whereas SDMA und ADMA levels further decreased in children. SDMA and ADMA are higher in children with CKD independent of GFR (mean factor 1.92 and 1.38, respectively, *p* < 0.001 for both), and SDMA is strongly correlated with creatinine concentration in children with CKD (Spearman’s rho 0.74, *p* < 0.001). We provide guanidino compound levels in a large sample covering all paediatric age groups for the first time. Our data can be used to assess the role of guanidino compounds such as hArg in disease states, i.e. cerebro- and cardiovascular disorders in childhood and adolescence.

## Introduction

Nitric oxide (NO) functions as potent vasorelaxant, inhibitor of platelet aggregation and neurotransmitter. The main substrate for NO synthase is L-arginine (L-Arg) which results from the urea cycle in the kidney. Guanidino compounds with structural similarity can alter L-Arg bioavailability. Among guanidino compounds, asymmetric dimethylarginine (ADMA), symmetric dimethylarginine (SDMA) and L-homoarginine (L-hArg) have emerged as potential modulators of NO metabolism. Whereas ADMA serves as endogenous NO inhibitor, SDMA inhibits cellular L-Arg uptake, and L-hArg may act as substrate for NO synthetase and increase L-Arg bioavailability by inhibiting arginase (Grosse et al. 2020).

High ADMA and SDMA, but low L-hArg concentrations have been associated with cardio- and cerebrovascular events and mortality (Wanby et al. 2006; Pilz et al. 2011; Grosse et al. 2020). In both population-based and clinical cohorts, ADMA and SDMA concentrations are correlated with cardiovascular risk burden, e.g. CHA2DS2-VASC (Cordts et al. 2019; Grosse et al. 2019). Furthermore, patients with acute ischaemic stroke or myocardial infarction have higher ADMA and SDMA concentrations compared with healthy controls (Schulze 2010, Lüneburg 2012, Jud 2018). Lower L-hArg concentrations are associated with increased adverse events after stroke or cardiovascular events (März et al. 2010; Atzler et al. 2015). Moreover, a causative role for L-hArg in stroke has been demonstrated in mouse models lacking endogenous L-hArg synthesis by arginine:glycine amidinotransferase (AGAT) (Choe et al. 2013).

In addition to cardiovascular function and disease, numerous studies have linked these three guanidino compounds with kidney function. High SDMA and low L-hArg concentrations have been repeatedly shown to correlate with glomerular filtration rate (GFR) (Brooks et al. 2009; Schwedhelm and Böger 2011; Snauwaert et al. 2018a). Especially SDMA is a reliable blood-based marker for early kidney impairment in humans and animals (Schwedhelm and Böger 2011). In adults, L-hArg levels decrease with age, whereas ADMA and SDMA levels increase (Atzler et al. 2014). Of note, in children and adolescents, ADMA concentrations were found to decrease with age, whereas no clear correlation of SDMA with age has been observed (Lücke et al. 2007; Jaźwińska-Kozuba et al. 2013). On the other hand, L-hArg increases from 3 to 18 years of age (Jaźwińska-Kozuba et al. 2013). Interestingly, L-hArg levels also increase during pregnancy from first to third trimester and correlate with flow-dilated vasodilatation (FMD) (Valtonen et al. 2008). However, concentrations of hArg, SDMA and ADMA and their precursors have not been measured systematically in larger cohorts of children younger than 3 years. Given the complex role of guanidino compounds in NO metabolism and cardiovascular disorders, we examined the concentrations of hArg, SDMA and ADMA in neonates, children and adolescents including paediatric patients with chronic kidney disease (CKD).

## Methods

### Study design, ethical approval and patient consent

This study is a cross-sectional single-centre cohort at the department of paediatrics at the University Medical Center Hamburg-Eppendorf. 141 patients without evidence of impaired renal function and cardio- or cerebrovascular disorder were classified according to their age into 5 subgroups (0–28 days; 1–11 months; 1–6 years; 7–12 years; 13–17 years). Creatinine concentrations were measured in 123 of 141 patients (creatinine levels were not routinely measured in healthy newborns). In addition, 57 paediatric patients with chronic kidney disease (CKD) were included into a separate cohort. GFR was calculated using the Schwarz formula (Schwartz et al. 1976).

The study protocol was approved by the Ethics Committee of the Hamburg Board of Physicians (PV5279). The investigation was conducted in accordance with the Declaration of Helsinki.

### Laboratory measurements

The laboratory measurements of guanidino compounds were obtained from blood samples stored at − 80 °C, and processed as previously described with minor modifications (Atzler et al. 2011; Cordts et al. 2015). In brief, 25 μl of EDTA plasma, calibrator or quality control sample was subjected to protein precipitation with 100 μl methanol containing stable isotope-labelled internal standards. Residues were derivatised to their butyl ester derivatives and reconstituted samples were separated on an AQUITY UPLC BEH C18 1.7 µm (2.1 × 50 mm) column (Waters, Eschborn, Germany) using an elution gradient of the two mobile phases (A) 0.1% formic acid in water and (B) 0.1% formic acid in acetonitrile at a flow rate of 0.4 mL/min over 2.6 min. Quantification was performed with a Xevo Triple Quadrupole Mass Spectrometer (Waters) with positive electrospray ionisation in the multiple reaction mode (MRM). Peak area ratios of analyte and internal standard were calculated for calibration (four levels), quality control (QC-low and -high), and study samples and used for quantification. Quality control samples were accepted below 15% relative standard deviation (RSD).

### Statistical analysis

Continuous variables are given as mean ± standard deviation (SD) if normally distributed, otherwise as median [25th–75th percentiles], and categorical variables are given as numbers (percentage) of participants. Relationships with continuous variables were assessed by Spearman correlation. Statistical comparisons of age groups were made by Kruskal–Wallis with Bonferroni post hoc test or Chi^2^ test as appropriate. For statistical comparisons of control children and children with CKD, we used Mann–Whitney *U *test. The independent association between control and CKD patients with hArg, SDMA and ADMA was determined by multivariable linear regression analyses (mean factor and 95% confidence interval, CI). For unadjusted and adjusted linear regression analyses, we calculated beta coefficients for different models: unadjusted (model 1), adjusted for age and weight (model 2), and adjusted for age, weight and GFR (model 3). A *p* value < 0.05 was considered statistically significant. Statistical analysis was performed with IBM SPSS Statistics (version 22, IBM Corp., Armonk, NY) and GraphPad Prism (version 5 for Windows, La Jolla, USA).

### Data availability statement

Deidentified patient data, related documents such as study protocol and statistical analysis plan will be shared by request from any qualified investigator for 3 years after the date of publication.

## Results

**Fig. 1:**
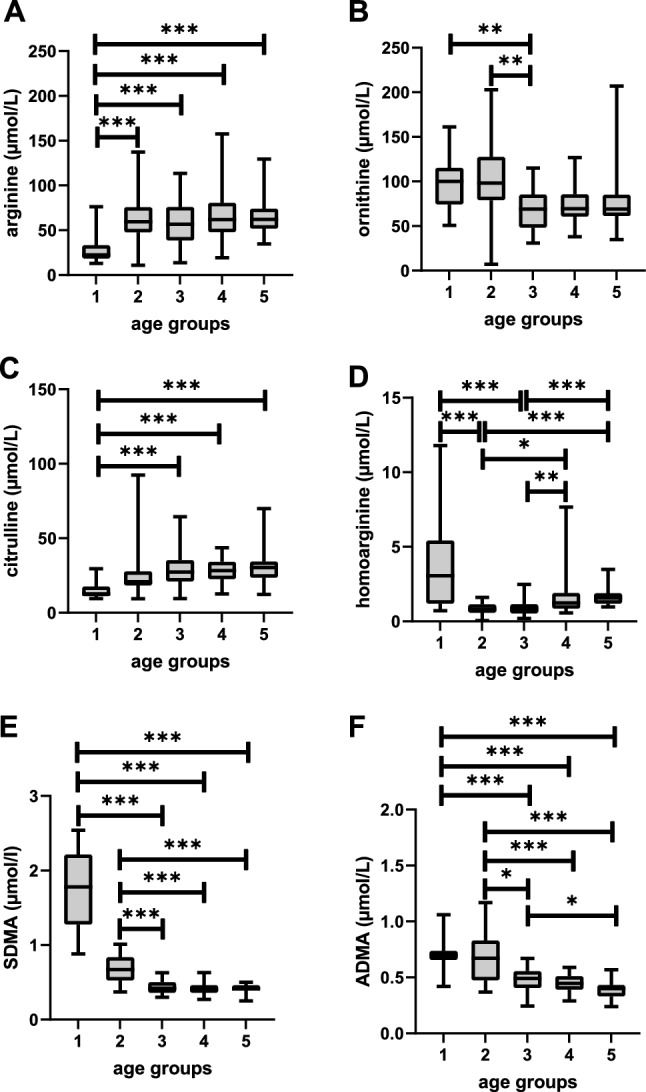
L-Arg, ornithine, citrulline, hArg, SDMA and ADMA plasma concentrations in children of age groups 1–5. Data are displayed as boxplots with median and interquartile range. Statistical analysis was performed using Kruskal–Wallis with Bonferroni post hoc test (*p < 0.05, **p < 0.01 and ***p < 0.001)

In our baseline cohort, 141 children and adolescents across age groups 1–5 without CKD or evidence of cerebro- or cardiovascular disorder were included (Table 1). Comparison between age groups revealed significant differences for haemoglobin, creatinine, GFR, L-Arg, citrulline, ornithine, hArg, SDMA and ADMA concentrations (Table 1). In post hoc analysis, neonates (age group 1, i.e. neonates < 29 days) had lower L-Arg and citrulline, but higher ornithine, hArg, SDMA and ADMA plasma levels compared with age groups 3–5, i.e. 1 year and older (Fig. 1). ADMA and SDMA plasma levels were also higher in children of age group 2 (1–11 months) compared with older age groups, whereas hArg dropped from high levels in age group 1 and then gradually increased from age group 2–5 (Fig. 1).

**Table 1:** Baseline characteristics of our cohort including 141 patients without CKD subdivided into 5 age groups

Characteristics	Age group 1 (< 29 days, *n* = 18)	Age group 2 (1–11 month, *n* = 21)	Age group 3 (1–5 years, *n* = 41)	Age group 4 (6–11 years, *n* = 30)	Age group 5 (12–17 years, *n* = 31)	*p* value
Female, *n*	6 (33.3%)	6 (28.6%)	21 (51.2%)	15 (50.0%)	19 (61.3%)	0.576
Weight, kg	3.3 (0.6)	7.2 (1.6)	15.9 (4.7)	38.2 (14.6)	60.7 (15.5)	< 0.001***
Hb, g/dl	17.3 [15.9, 20.1]^+^	11.9 [10.8, 12.8]	12.1 [11.5, 12.8]	13.2 [12.6, 13.9]	13.9 [12.5, 14.9]	< 0.001***
Creatinine, mg/dl	0.39 [0.24, 0.74]^#^	0.25 [0.18, 0.30]	0.30 [0.24, 0.36]	0.51 [0.46, 0.57]	0.66 [0.60, 0.75]	< 0.001***
GFR, ml/min	52 [29, 72]^#^	113 [95, 155]	145 [124, 182]	122 [110, 139]	106 [98, 122]	< 0.001***
Lysine, µmol/L	162 [137, 193]	144 [121, 192]	136 [106, 170]	170 [143, 191]	160 [140, 182]	0.056
Arginine, µmol/L	22 [18, 33]	59 [48, 76]	57 [39, 76]	62 [48, 81]	62 [52, 74]	< 0.001***
Citrulline, µmol/L	12.7 [11.2, 17.6]	20.8 [18.4, 27.9]	27.3 [21.1, 35.3]	28.3 [22.5, 34.2]	30.3 [23.6, 34.4]	< 0.001***
Ornithine, µmol/L	100.1 [74.2, 115]	98.1 [79.1, 127.6]	69.0 [48.1, 85.3]	69.6 [60.8, 85.5]	69.0 [61.2, 85.0]	< 0.001***
hArg, µmol/L	3.06 [1.19, 5.42]	0.81 [0.58, 1.13]	0.83 [0.53, 1.15]	1.23 [0.85, 1.90]	1.57 [1.20, 1.88]	< 0.001***
SDMA, µmol/L	1.78 [1.27, 2.21]	0.67 [0.53, 0.83]	0.42 [0.36, 0.50]	0.40 [0.36, 0.46]	0.41 [0.39, 0.46]	< 0.001***
ADMA, µmol/L	0.70 [0.65, 0.74]	0.67 [0.47, 0.83]	0.40 [0.49, 0.55]	0.45 [0.39, 0.51]	0.40 [0.33, 0.43]	< 0.001***

Data are given as number (%), mean (SD) or median [interquartile range] as appropriate

*ADMA* asymmetric dimethylarginine, *Hb* haemoglobin, *hArg* homoarginine, *SDMA* symmetric dimethylarginine

(^#^available from 7/18, ^+^available from 9/18)

Given the obvious difference of metabolite kinetics in neonates, we analysed age groups 2–5 separately from age group 1. In children of age groups 2–5, age and creatinine were positively associated with hArg, whereas ADMA and SDMA showed a negative correlation (Table 2). hArg, SDMA and ADMA were positively correlated with ornithine levels (Table 2). Similarly, positive correlations were found for hArg and ADMA with L-Arg and L-lysine, whereas only hArg revealed a positive association with L-citrulline (Table 2). In contrast, hArg and SDMA concentrations strongly and negatively decreased with age in neonates. This is in accordance with high maternal hArg levels during late pregnancy (Table 3) (Valtonen et al. 2008).

**Table 2:** Spearman correlation analysis of hArg, ADMA and SDMA plasma levels with age and laboratory parameters in children of age groups 2–5 (1 month–17 years)

	Parameter	correlation coefficient rho	P value
hArg	Age	0.597	< 0.001***
	Creatinine	0.530	< 0.001***
	Lysine	0.554	< 0.001***
	Arginine	0.445	< 0.001***
	Citrulline	0.308	0.001**
	Ornithine	0.241	0.007**
SDMA	Age	-0.397	< 0.001***
	Creatinine	-0.144	0.120
	Lysine	-0.018	0.841
	Arginine	0.068	0.456
	Citrulline	0.033	0.714
	Ornithine	0.245	0.006**
ADMA	Age	-0.550	< 0.001***
	Creatinine	-0.455	< 0.001***
	Lysine	0.313	< 0.001***
	Arginine	0.392	< 0.001***
	Citrulline	0.082	0.366
	Ornithine	0.492	< 0.001***

**p < 0.01, ***p < 0.001

**Table 3:** Spearman correlation analysis of age with ornithine, L-Arg, lysine, hArg, ADMA and SDMA plasma levels in children of age group 1 (< 29 days)

	Parameter	Correlation coefficient rho	*p *value
Age	Ornithine	0.678	0.002**
	Lysine	0.284	0.254
	L-Arg	0.550	0.018*
	Citrulline	0.117	0.643
	hArg	− 0.831	< 0.001***
	SDMA	− 0.659	0.003**
	ADMA	0.163	0.518

*p < 0.05, **p < 0.01 and ***p < 0.001

We compared 123 children with normal renal function and 57 children with CKD. As expected, children with CKD had lower haemoglobin and higher creatinine plasma levels. In addition, L-citrulline, SDMA and ADMA plasma levels were increased compared with control children (Table 4). In linear regression analysis, SDMA and ADMA levels were 1.92-fold and 1.38-fold higher in CKD compared with control children in models also adjusted for age, weight, and GFR (Table 5). Consistently, ADMA and SDMA were negatively and hArg was positively correlated with age in CKD children (Table 6). A positive correlation was also found for ADMA and SDMA with citrulline (Table 6). Interestingly, only SDMA levels were correlated with creatinine concentrations in CKD children, whereas hArg and ADMA were not (Table 6).

**Table 4:** Comparison of clinical and laboratory parameters between control and CKD children

	Control (*n* = 123)	CKD (*n* = 57)	*p *value
Age, months	87 [31, 159]	104 [39.5, 172]	0.195
Weight, kg	25 [13, 50]	29 [13, 51]	0.498
Hb, g/dl	12.7 [11.9, 13.5]	11.6 [10.6, 12.8]	< 0.001***
Creatinine, mg/dl	0.43 [0.27, 0.60]	1.41 [0.84, 3.53]	< 0.001***
Lysine, µmol/L	129 [152, 188]	146 [119, 194]	0.513
Arginine, µmol/L	61.6 [46.3, 75.9]	67.3 [56.7, 86.0]	0.013*
Citrulline, µmol/L	27.8 [21.0, 33.6]	56.4 [43.2, 80.2]	< 0.001***
hArg, µmol/L	1.10 [0.73, 1.51]	0.94 [0.63, 1.31]	0.089
SDMA, µmol/L	0.43 [0.37, 0.52]	1.28 [0.77, 3.05]	< 0.001***
ADMA, µmol/L	0.45 [0.39, 0.54]	0.66 [0.58, 0.78]	< 0.001***

Data are given as median [IQR]

Comparison of two independent groups with Mann–Whitney *U* test (*p < 0.05 and **p < 0.01)

**Table 5:** Linear regression analysis of hArg, ADMA and SDMA plasma levels in control and CKD children

		hArg		SDMA		ADMA	
	Model	Mean factor (95% CI)	*p *value	Mean factor (95% CI)	*p *value	Mean factor (95% CI)	*p *value
Control vs CKD	1	0.82 (0.67, 1.00)	0.055	3.43 (2.86, 4.11)	< 0.001***	1.43 (1.30, 1.57)	< 0.001***
	2	0.76 (0.64, 0.90)	0.002**	3.61 (3.05, 4.28)	< 0.001***	1.50 (1.38, 1.61)	< 0.001***
	3	0.78 (0.60, 1.00)	0.053	1.92 (1.56, 2.37)	< 0.001***	1.38 (1.23, 1.55)	< 0.001***

Linear regression analysis with beta coefficients (95% confidence interval). _Model 1: unadjusted; model 2: adjusted for age and weight; model 3: adjusted for age, weight and GFR

**Table 6:** Spearman correlation analysis of hArg, ADMA and SDMA plasma levels with age and laboratory parameters in CKD children

	Parameter	Correlation coefficient rho	*p *value
hArg	Age	0.504	< 0.001***
	Creatinine	− 0.137	0.309
	Lysine	0.314	0.017*
	Arginine	0.289	0.029*
	Citrulline	− 0.157	0.245
	Ornithine	0.111	0.410
SDMA	Age	− 0.385	0.003**
	Creatinine	0.740	< 0.001***
	Lysine	− 0.079	0.559
	Arginine	− 0.031	0.819
	Citrulline	0.799	< 0.001***
	Ornithine	− 0.063	0.639
ADMA	Age	− 0.498	< 0.001***
	Creatinine	0.234	0.080
	Lysine	0.215	0.108
	Arginine	0.218	0.103
	Citrulline	0.478	< 0.001***
	Ornithine	0.170	0.206

^*^p < 0.05, **p < 0.01 and ***p < 0.001

Other factors than CKD have also been shown to interfere with guanidino compound metabolism (Hanusch et al. 2020). We, therefore, analysed the effects of either inflammatory disorders, immunosuppressive medication or vitamin D supplementation on guanidino compound levels in our control cohort (Table 7). Vitamin D supplementation had no significant effect on SMDA, ADMA and hArg plasma levels. Both inflammatory disorders and immunosuppressive medication, however, were associated with lower SDMA levels, but not with altered hArg or ADMA levels.

**Table 7:** Comparison of hArg, SDMA and ADMA plasma levels in patients with and without chronic inflammation, immunosuppressive medication or vitamin D intake

	No chronic inflammation (*n* = 113)	Chronic inflammation (*n* = 28)	*p *value
hArg, µmol/L	1.11 [0.76, 1.58]	1.30 [0.83, 2.02]	0.360
SDMA, µmol/L	0.47 [0.39, 0.65]	0.39 [0.36, 0.48]	0.003**
ADMA, µmol/L	0.50 [0.40, 0.64]	0.42 [0.40, 0.49]	0.081
	No immunosuppressive medication (*n* = 121)	Immunosuppressive medication (*n* = 20)	*p *value
hArg, µmol/L	1.13 [0.75, 1.63]	1.43 [0.89, 1.87]	0.413
SDMA, µmol/L	0.46 [0.39, 0.63]	0.39 [0.36, 0.48]	0.017*
ADMA, µmol/L	0.48 [0.40, 0.64]	0.44 [0.40, 0.51]	0.134
	No Vitamin D (*n* = 93)	Vitamin D (*n* = 48)	*p *value
hArg, µmol/L	1.24 [0.78, 1.83]	0.98 [0.79, 1.49]	0.101
SDMA, µmol/L	0.45 [0.39, 0.56]	0.49 [0.37, 0.68]	0.511
ADMA, µmol/L	0.47 [0.39, 0.59]	0.47 [0.40, 0.67]	0.234

Data are given as median [IQR]. Comparison of two independent groups with Mann–Whitney *U* test (*p < 0.05 and **p < 0.01)

## Discussion

In this study, we provide the first dataset of guanidino compound levels including all paediatric age groups including neonates as well as children with CKD. Our main findings are that (1) neonates have higher hArg, SDMA and ADMA plasma concentrations compared with children older than 12 months, (2) hArg levels increase, whereas SDMA und ADMA levels decrease in children from the age of 2 months on, (3) SDMA and ADMA are higher in children with CKD independent of GFR and (4) only SDMA but not ADMA and hArg are strongly correlated with creatinine concentration children with in CKD.

To date, there are no reference values available for guanidino compounds in individuals younger than 18 years. Several studies have reported guanidino compound levels including L-hArg, ADMA and SDMA in paediatric healthy control subjects. To our knowledge, the largest cohort consisted in 78 children from the age of 5–17 years (Hanusch et al. 2020). Other studies included younger children but smaller overall numbers (Snauwaert et al. 2018a). However, guanidino compound levels especially in neonates have not been analysed before. Our control cohort includes 141 subjects of distinct paediatric age groups including neonates which allows us to identify correlations of guanidino compound levels with different parameters such as age and precursor metabolite concentrations. Our results are in the same range as previously published average concentrations for the relevant guanidino metabolites L-Arg (44–93 µmol/l) (Buck et al. 2017; Hanusch et al. 2020), L-hArg (1.2–1.8 µmol/l) (Hörster et al. 2015; Hanusch et al. 2022), SDMA (0.4–0.7 µmol/l) (Brooks et al. 2009; Andrade et al. 2015), and ADMA (0.6–0.9 µmol/l) (Langen et al. 2015; Buck et al. 2017) in children and adolescents.

In neonates, hArg and SDMA levels steadily decreased during the first 28 days after birth. In our cohort, hArg levels in neonates were 3.06 [1.19, 5.42] µmol/l and therefore much higher than in children and adolescents. Interestingly, Valtonen and colleagues have shown that L-hArg concentrations are significantly increased during the second and third trimester in pregnant women reaching concentrations up to 5 µmol/l (Valtonen et al. 2008). Therefore, high hArg concentrations in neonates are likely not due to endogenous synthesis in neonates but result from elevated maternal hArg. Given that hArg levels continuously increase from age group 2 with median levels of 0.8 µmol/l to age group 5 with median levels of 1.57 µmol/l, endogenous production seems to be low in early development and does steadily evolve during childhood. In accordance, a previous study in children and adolescents revealed increasing L-hArg in the age range of 3–18 years (Jaźwińska-Kozuba et al. 2013).

SDMA and ADMA both result from protein degradation by N-methyl protein transferases. Similar to hArg, SDMA levels drop during the first weeks of life from elevated levels to a baseline around 0,4 µmol/l and do not increase again during childhood and adolescence. SMDA is excreted almost exclusively via the kidney, and high SDMA levels in early development might either indicate low renal filtration rate in newborns, increased protein degradation or exogenous supply during pregnancy. In a similar but less pronounced fashion, ADMA concentrations decline from slightly elevated levels after birth to around 0,5 µmol/l in infancy, but continuously decrease during childhood and adolescence. ADMA is metabolised in liver and kidney, and excreted via the kidney to a smaller extent (Chen et al. 2012). ADMA levels, thus, might also mirror protein turnover which is high during periods of rapid growth during early development and steadily declines during development.

The high levels of all three guanidino compounds ADMA, SDMA and hArg in neonates are puzzling, and the physiological consequences are not entirely clear. ADMA and SDMA are negatively correlated with cardiac function and circulation in adults (Wanby et al. 2006; Grosse et al. 2020). hArg, on the other hand, exerts positive effects on cardiac and vascular function as well as overall mortality (Atzler et al. 2017; Faller et al. 2018; Rodionov et al. 2019; Schwedhelm et al. 2020). Following low endogenous L-hArg production due to immature renal synthesis in the developing foetus and neonates, maternal supply of high levels of L-hArg might thus be protective to counterbalance excessive SDMA/ADMA levels during this early developmental period.

Guanidino compounds are in part synthetised and excreted via the kidney. Consequently, the levels of ADMA, SDMA and L-hArg have been shown to be correlated with renal function in adults (Drechsler et al. 2013; Tomaschitz et al. 2014; Snauwaert et al. 2018a). Similar results have been published for SDMA and ADMA in paediatric patients suffering from CKD, but L-hArg levels have not been previously analysed in detail (Brooks et al. 2009; Snauwaert et al. 2018a, b). In accordance with previous studies, our data confirm the strong correlation of SDMA and to a lesser extent ADMA with renal function. Especially SDMA has been proposed as a sensitive surrogate marker for GFR (Schwedhelm and Böger 2011). Our data demonstrate a strong age dependence during the first months which might limit its use during this age group. However, we did not analyse the correlation of SDMA and GFR in newborns and toddlers in detail. With regard to hArg, we only found a trend, but not a clear correlation between plasma concentrations and CKD possibly due to the relatively preserved renal function in our CKD group and small number of individuals.

In paediatric patients, guanidino compounds have previously been analysed in a number of conditions such as growth retardation, airway inflammation, atopic diseases or muscular dystrophy (Hörster et al. 2015; Langen et al. 2015; Hanusch et al. 2020, 2022). Elevated L-hArg levels have been found in short stature children, for example (Langen et al. 2015). Minor alterations in the Arg/NO pathway have been described in patients with inflammatory diseases such as cystic fibrosis, but antibiotic treatment led to a significant reduction in local L-hArg in sputum (Hanusch et al. 2022). In addition, chronic treatment with steroids did not affect guanidino compound levels in a cohort of Duchenne patients (Hörster et al. 2015). In our sample, we detected reduced SDMA levels in both inflammatory disorders and immunosuppressive treatment, whereas ADMA and hArg plasma levels remained unaffected. As SDMA is produced by N-methyl protein transferases during protein degradation, decreased levels following acute antibiotic treatment might reflect reduction in protein turnover. Reduced SDMA levels under immunosuppressive medication in our cohort of patients might also be explained by reduced protein turnover. The reason for lower SDMA levels in inflammatory disorders, however, remains unclear.

In summary, we provide guanidino compound levels of all paediatric age groups in a relatively large clinical sample. We show for the first time that hArg and SDMA plasma concentrations are highest in neonates compared with older children. Furthermore, our data confirm that SDMA is a valuable marker to distinguish and quantify CKD in children. Our data can be used to assess the role of guanidino compounds such as hArg in other disease states in the future, i.e. in cerebro- and cardiovascular disorders during childhood and adolescence.

## Data Availability

Data generated during this study are available from the corresponding author on request.

## Abbreviations

ADMA: Asymmetric dimethylarginine
CKD: Chronic kidney disease
GFR: Glomerular filtration rate
L-Arg: L-Arginine
hArg: Homoarginine
SDMA: Symmetric dimethylarginine
